# The DRESS/HLH Overlap Syndrome: A Systematic Review of Clinical Features, Triggers, and Outcomes

**DOI:** 10.3390/jcm15197383

**Published:** 2026-09-23

**Authors:** Dorde Jevtic, Igor Dumic

**Affiliations:** 1 Division of Allergy and Immunology, Icahn School of Medicine at Mount Sinai, New York, NY 10029, USA; 2 Department of Hospital Medicine, Mayo Clinic Health System, Eau Claire, WI 54703, USA; dumic.igor@mayo.edu; 3 Division of Hospital Medicine, Department of Medicine, Lahey Clinic, Burlington, MA 01805, USA

**Keywords:** DRESS syndrome, hemophagocytic lymphohistiocytosis, drug hypersensitivity, hyperinflammatory syndrome, overlap syndrome, systematic review

## Abstract

**Background/Objectives**: Drug reaction with eosinophilia and systemic symptoms (DRESS) syndrome and hemophagocytic lymphohistiocytosis (HLH) are life-threatening conditions characterized by immune dysregulation. Several reports have identified an overlap between these entities; however, clinical characteristics, triggers, and outcomes remain poorly defined. The objective of this study was to characterize the clinical features and outcomes of DRESS/HLH overlap syndrome. **Methods**: We conducted a PRISMA-guided systematic review of PubMed, Embase, and MEDLINE. Studies were included if they reported cases meeting criteria for DRESS syndrome and HLH. **Results**: Thirty-six patients were included, 75% female and 27.8% pediatric. Initial misdiagnosis occurred in 30.6% and was associated with higher mortality (45.5% vs. 20.0%). Antibiotics were the most frequent triggers (36.1%) and 11.1% of patients had no trigger identified. Mean latency was 27.9 days for DRESS syndrome and 35 days for HLH, with shorter latency in antibiotic-associated cases. DRESS syndrome and HLH most commonly occurred sequentially (n = 22, 61.1%) and in 52.8% of cases DRESS preceded HLH. Fever and rash were present in 88.9%. Laboratory abnormalities included hyperferritinemia (91.7%), eosinophilia (77.8%), anemia (75.0%), and thrombocytopenia (75.0%). Hepatic involvement was nearly universal (94.4%), followed by renal involvement (36.1%). Viral reactivation was observed in 38.9%, multiorgan failure in 36.1%, and ICU admission in 19.4% of cases. Mortality was 27.8% and higher in steroid-refractory cases and those with multiorgan failure. Treatment included corticosteroids (97.2%), with IVIG (33.3%), etoposide (13.9%), and cyclosporine (5.6%) used in severe cases. **Conclusions**: DRESS-HLH overlap represents a severe hyperinflammatory syndrome with high mortality. Early recognition and escalation of therapy are essential to improve outcomes. This is the largest and most uniformly characterized cohort of DRESS/HLH overlap reported to date, and the first to stratify mortality by diagnostic accuracy, temporal pattern, corticosteroid responsiveness, and multiorgan failure. Because the syndrome is rare and the evidence base consists almost entirely of single-case reports, multicenter collaboration and a prospective registry are needed to define incidence and optimal therapy.

## 1. Introduction

Drug reaction with eosinophilia and systemic symptoms (DRESS) syndrome is a severe, potentially life-threatening hypersensitivity reaction characterized by fever, cutaneous eruption, hematologic abnormalities, and sometimes internal organ involvement [1,2]. Its pathogenesis is complex and involves a combination of drug-specific immune responses, viral reactivation, and dysregulated T-cell activation [3,4]. Hemophagocytic lymphohistiocytosis (HLH) is a hyperinflammatory syndrome driven by uncontrolled immune activation and cytokine release, which may be primary (familial) or secondary to infections, malignancy, autoimmune disease, or medications [5,6,7].

Although DRESS syndrome and HLH are distinct clinical entities, evidence suggests a potential overlap, with DRESS syndrome acting as a trigger for secondary HLH [8]. These conditions may represent points along a shared immunopathologic spectrum in the context of drug-induced immune dysregulation with or without viral reactivation [8]. DRESS/HLH overlap syndrome poses significant diagnostic and therapeutic challenges, given the substantial overlap in clinical and laboratory features and the differing management strategies required for each condition [1,5,8].

Both syndromes can mimic other febrile diseases with multi-system involvement and are potentially life-threatening, and delays in diagnosis and treatment are associated with increased mortality [1,5]. Accordingly, clinicians should maintain a high index of suspicion for these conditions and their potential overlap. The differential diagnosis is broad and includes sepsis and severe bacterial or viral infection, primary Epstein–Barr virus and cytomegalovirus infection, adult-onset Still disease and systemic juvenile idiopathic arthritis, lymphoma and leukemia, systemic lupus erythematosus and vasculitis, Stevens–Johnson syndrome/toxic epidermal necrolysis, acute generalized exanthematous pustulosis, and drug-induced liver injury, each of which may present with fever, rash, cytopenias, and organ dysfunction. Given the rarity of this entity, large prospective studies are lacking, and available retrospective data remain limited. No dedicated registry or multicenter cohort of DRESS/HLH overlap exists, and the published evidence consists almost entirely of individual case reports, so that no single center is likely to accumulate sufficient experience to characterize the syndrome on its own. Therefore, the aim of this study was to synthesize evidence from published case reports to better characterize the clinical features, triggers, and outcomes of DRESS/HLH overlap syndrome.

## 2. Materials and Methods

We conducted a systematic review in accordance with the Preferred Reporting Items for Systematic Reviews and Meta-Analyses (PRISMA) guidelines, and a PRISMA flow diagram was generated to illustrate the study selection process (Figure 1). A comprehensive literature search for cases of DRESS and HLH was performed using PubMed, Embase, and MEDLINE databases from inception through March 2026. Reference lists of included cases were manually screened to identify additional relevant publications. Our review included a total of 28 articles and 5 conference abstracts, amounting to 36 individual cases (Figure 1) (Supplementary Material: PRISMA checklist) [9,10,11,12,13,14,15,16,17,18,19,20,21,22,23,24,25,26,27,28,29,30,31,32,33,34,35,36,37,38,39,40,41].

The following search strategy, adapted to the syntax of each database, was applied across all sources: (“DRESS syndrome” OR “drug reaction with eosinophilia and systemic symptoms”) AND (“hemophagocytic lymphohistiocytosis” OR “HLH” OR “hemophagocytic syndrome” OR “macrophage activation syndrome”). Searches were restricted to English-language publications and were last conducted on 1 March 2026, with no additional date or publication-type filters applied. This review was not prospectively registered in PROSPERO or another public registry. The search was restricted to English-language publications, as our team was unable to verify the reported diagnostic criteria and clinical data of non-English reports; four potentially eligible non-English reports were identified during full-text screening but were not included.

Studies were included if they reported cases of DRESS/HLH overlap with sufficient clinical detail to confirm both diagnoses, either defined by a RegiSCAR score ≥ 5 and an HScore ≥ 168 or by reporting compatible clinical and laboratory features. RegiSCAR and HScore values were taken as reported by the original case authors; these scores were not independently calculated from the underlying clinical and laboratory data. Studies were excluded if the diagnosis of either condition was uncertain or not supported by established diagnostic criteria, or if they were review articles or editorials or lacked primary data for verification of clinical or laboratory values. When duplicate cases were identified, the most comprehensive report was included. Included studies were not grouped into separate syntheses; all eligible cases were analyzed together in a single descriptive synthesis. All studies excluded after full-text review were excluded for the reasons summarized in Figure 1 (unconfirmed diagnosis of DRESS syndrome or HLH, duplicate reporting, or non-English language); no additional reports that appeared potentially eligible were excluded for other reasons.

Two independent reviewers screened titles and abstracts, followed by full-text review to determine eligibility, with discrepancies resolved by consensus. Data were extracted using a standardized form and included patient demographics, suspected triggers, temporal relationship between DRESS syndrome and HLH, clinical and laboratory features, treatment approaches, and outcomes. Implicated medications were recorded both individually and grouped into major categories: antibiotics, antiepileptic drugs, and other agents. Cases were classified as ‘concurrent’ or ‘sequential’ based on how the temporal relationship was reported in the source publication; this was stated explicitly in some reports and inferred from the clinical narrative in others when not explicitly stated. Sequential cases were further defined as DRESS syndrome preceding HLH, HLH preceding DRESS syndrome, DRESS syndrome with initial improvement followed by HLH, or HLH with initial improvement followed by DRESS syndrome. Steroid-refractory disease was defined as clinical or laboratory worsening over the course of corticosteroid therapy. Due to variability in reporting across case reports and potential inconsistencies during data extraction, missing binary variables were conservatively coded as absent to maintain internal consistency.

Each included case was independently appraised for quality using the JBI Critical Appraisal Checklist for Case Reports [42], which evaluates the clarity of reporting of patient demographics, history, clinical presentation, diagnostic assessment, treatment, post-intervention outcome, and adverse events; all cases were rated as appropriate for inclusion, though several had incomplete reporting on RegiSCAR and HScore values as described above. Descriptive statistics were used to summarize clinical characteristics and outcomes.

## 3. Results

The cohort included 36 patients with confirmed DRESS syndrome and HLH, comprising 9 male (25.0%) and 27 female patients (75.0%), with mean ages of 41.6 ± 21.7 and 36.1 ± 21.1 years, respectively. Pediatric patients represented 27.8% of the cohort (n = 10), with a comparable sex distribution (males: n = 3, 30.0%; females: n = 7, 70.0%) (Table 1).

The initial diagnosis of DRESS syndrome, HLH, or DRESS/HLH overlap was correct in 25 (69.4%) cases. Among patients with an initially incorrect diagnosis, the majority were presumed to have a viral or bacterial infection (n = 9, 81.8%). The mean latency from drug exposure to the onset of DRESS syndrome was 27.9 days, while HLH developed at a mean of 35.0 days. Notably, antibiotic-associated cases demonstrated a shorter latency to both DRESS and HLH onset compared to non-antibiotic triggers (DRESS syndrome: 16.9 vs. 33.2; HLH: 24.8 vs. 39.9 days). DRESS syndrome and HLH most commonly occurred sequentially, observed in 22 cases (61.1%), of which DRESS preceding HLH was most common (n = 19, 52.8%) (Table 2). Mortality was higher among patients with an initially incorrect diagnosis (45.5%) compared to those correctly diagnosed (20.0%).

Antibiotics were the most frequently implicated class of medications, accounting for 13 cases (36.1%) (Table 1). However, the most commonly implicated individual drugs were sulfasalazine (n = 5, 13.9%) followed by allopurinol (n = 4, 11.1%), carbamazepine (n = 3, 8.3%), and piperacillin–tazobactam, which was identified either as a definitive (n = 2, 5.6%) or suspected (n = 2, 5.6%) trigger. In 4 cases (11.1%), an antibiotic was suspected but not specified, and in 4 cases (11.1%), the culprit medication was unknown. Less common medications included phenytoin, leflunomide, hydroxychloroquine, teicoplanin, lamotrigine, trimethoprim–sulfamethoxazole, minocycline, tocilizumab, cefpodoxime, valproic acid, amoxiclav, ceftazidime, and phenobarbital (each n = 1, 2.8%). In one case, the culprit was a nutritional supplement, Boswellia serrata.

The most common presenting symptoms were fever and rash (each n = 32, 88.9%). Other reported symptoms included abdominal pain, fatigue, and sore throat (each n = 4, 11.1%); jaundice and vomiting (each n = 3, 8.3%); and anorexia and arthralgia (each n = 2, 5.6%). Less frequent manifestations included dysphagia, headache, anuria, epistaxis, hematemesis, and altered mental status (each n = 1, 2.8%).

Hematologic, immunologic, and HLH-related laboratory findings are summarized in Table 1 alongside clinical features, visceral organ involvement, and viral reactivation subtypes.

Cutaneous involvement was most commonly described as a generalized maculopapular or morbilliform eruption, typically beginning on the trunk and spreading to the extremities and face. In several cases, the rash was extensive, involving 50–90% of body surface area or progressing to erythroderma. Facial edema was a frequent accompanying feature. Less common manifestations included pruritic eruptions, blistering or bullous lesions, exfoliative dermatitis, and, rarely, petechiae, ecchymoses, or hyperpigmented plaques.

Most commonly, patients were treated with corticosteroids (n = 35, 97.2%), which were tapered over a mean of 80.5 ± 63.9 days. Additional therapies are summarized in Table 1. Mortality was higher among patients who demonstrated clinical worsening despite initial corticosteroid therapy (47.4% vs. 5.9%).

The overall mortality rate in the cohort was 27.8% (n = 10/36) (Table 1). Multiorgan failure (MOF) was observed in 13 of 36 patients (36.1%) and was associated with worse outcomes. Mortality occurred in 53.8% (n = 7/13) of patients with MOF compared with 13.0% (n = 3/23) of those without. A total of 7 patients (19.4%) required admission to the intensive care unit (ICU), and 2 patients (5.6%) required transfer to a higher-acuity care facility. Secondary infections were documented in 4 patients (11.1%), most commonly superimposed bacterial infection and sepsis.

## 4. Discussion

In this study, DRESS/HLH overlap syndrome was observed more frequently in women, comprising three quarters of the cohort. Misdiagnosis was common, with nearly one-third of patients initially presumed to have a bacterial or viral infection which was associated with higher mortality. DRESS syndrome preceded HLH in 19 cases (53%). Viral reactivation was documented in 38% of cases, and antibiotics and sulfasalazine were the most frequently implicated medication class. The most common clinical and laboratory features included fever, eosinophilia, anemia, and thrombocytopenia, often accompanied by lymphadenopathy and splenomegaly. The liver and kidneys were the most frequently affected organs. Treatment strategies most commonly included systemic corticosteroids, IVIG, etoposide, and cyclosporine. MOF occurred in 36% of patients, and 19% required ICU admission. Patients with steroid-refractory disease had higher mortality, and the overall mortality rate in the cohort was 28%.

Our study highlights several important considerations and, to our knowledge, represents the first review to comprehensively and uniformly characterize the clinical features of patients presenting with DRESS/HLH overlap syndrome. As reflected in the included publications, interest in this entity has increased over the past decade, with a notable rise in reported cases between 2015 and 2025 compared to prior years (Figure 2). This trend likely reflects both a true increase in incidence and improved recognition and reporting of DRESS syndrome and HLH cases. With an increase in aging population and polypharmacy, the risk of idiosyncratic drug reactions continues to increase. Concurrently, growing awareness and an expanding body of literature have likely contributed to an improved recognition of these uncommon conditions.

Our findings both corroborate and extend the only prior synthesis of this entity, a review of 23 cases by Yang and colleagues [8]. The areas of agreement are substantial. Both cohorts describe a latency of roughly two to four weeks between drug exposure and the cutaneous eruption (a mean of 2.7 weeks in that series and 27.9 days in ours); both identify clinical deterioration despite initial therapy as the usual prompt for recognizing superimposed HLH, with 61% of their cases worsening before HLH was diagnosed a mean of 2.3 weeks after DRESS syndrome, mirroring the sequential pattern seen in 61.1% of our cases; and both identify viral reactivation and corticosteroid monotherapy as features of patients who fared poorly. Several findings differ. Whereas only 9 of 23 patients (39%) in the earlier review satisfied diagnostic criteria for both conditions and 5 met neither, all cases in the present cohort met RegiSCAR and HScore thresholds or were supported by explicitly reported compatible clinical and laboratory features, yielding a more homogeneous phenotype. Our cohort is also distinctly female-predominant (75%) and contains a substantial pediatric subgroup (27.8%) that was not separately characterized previously. Most importantly, the earlier review reported neither a pooled mortality estimate nor stratified outcomes, whereas our data provide an overall mortality of 27.8% together with stratification by initial diagnostic accuracy (45.5% vs. 20.0%), temporal pattern (31.8% sequential vs. 15.4% concurrent), corticosteroid responsiveness (47.4% vs. 5.9%), and multiorgan failure (53.8% vs. 13.0%). Finally, we identify antibiotics as the leading trigger class and show that antibiotic-associated cases have a shorter latency to both DRESS syndrome and HLH, an association not previously described in this population.

The novelty of this work therefore lies in three areas: it assembles the largest cohort of DRESS/HLH overlap reported to date and applies uniform inclusion criteria across all cases; it is the first to report mortality stratified by diagnostic accuracy, temporal pattern, corticosteroid responsiveness, and multiorgan failure; and it is the first to relate trigger class to latency within the overlap syndrome.

### 4.1. Demographics of Patients with DRESS/HLH Overlap Syndrome

Our cohort was predominantly female (75%), and one-quarter of cases were pediatric. This is similar to the established epidemiology of DRESS syndrome, which has been reported to occur more commonly in females [43], and differs from the epidemiology of HLH, which occurs slightly more frequently in adult male patients [44]. Both DRESS syndrome and secondary HLH typically affect adults aged 30 to 60, consistent with the age distribution observed in our study [2,44]. HLH is also well recognized in pediatric populations, most commonly as primary HLH due to inherited defects in cytotoxic function. In the existing literature, many pediatric cases did not undergo evaluation for primary (familial) HLH, despite this being the most common underlying etiology in this population. Once a medication trigger was identified, further investigation for alternative or underlying causes was often not pursued. However, emerging evidence supports a spectrum model of HLH, in which partial or heterozygous genetic variants may predispose patients to disease that is unmasked by external triggers such as infections or medications [45,46]. Future case reports should more consistently incorporate genetic evaluation and clearly document this assessment to improve characterization of underlying susceptibility, particularly in pediatric patients.

Similarly, reporting of race, ethnicity, and relevant pharmacogenetic data was limited and inconsistently described across published cases, further constraining interpretation. Geographic and ethnic variability has been noted in DRESS syndrome, exemplified by pharmacogenetic associations, with specific HLA alleles—such as HLA-B58:01 and HLA-A31:01—conferring increased risk [47,48]. In our cohort, one patient was identified as a carrier of HLA-B13:01 and developed DRESS syndrome following exposure to carbamazepine [15]. These findings further underscore the need for more comprehensive and standardized reporting of genetic and demographic data in future case reports.

### 4.2. Pathophysiology of DRESS/HLH Overlap Syndrome and Latency

The pathophysiology of DRESS syndrome and HLH is complex and incompletely understood. DRESS syndrome is a T cell-mediated delayed hypersensitivity reaction characterized by drug-specific immune activation, expansion of cytotoxic T cells, and frequent viral reactivation—most commonly HHV-6 and EBV—resulting in systemic inflammation and multiorgan involvement. It remains a clinical diagnosis, with the RegiSCAR scoring system used to classify cases as “no,” “possible,” “probable,” or “definite” [3,4,43]. HLH, in contrast, is a hyperinflammatory syndrome driven by impaired cytotoxic function of natural killer and CD8^+^ T cells, leading to uncontrolled macrophage activation and cytokine release [5,44,49].

While DRESS syndrome is always medication-induced, with or without associated viral reactivation, secondary HLH has traditionally been attributed to infectious, autoimmune, or malignant etiologies; however, in recent years, medication-induced HLH has increasingly been recognized [50,51,52]. Accordingly, both conditions may share overlapping triggers, including medications and viral reactivation, and their coexistence is not unexpected, with some cases likely representing different points along a shared disease continuum reflecting variable severity of immune dysregulation. This concept is further supported by a recent review proposing that lamotrigine and other anti-seizure medications may drive both HLH and drug-induced hypersensitivity syndrome/DRESS through a shared mechanism of medication-mediated T-cell activation [53].

In our cohort, DRESS syndrome developed several weeks after drug exposure (mean latency 27.9 days), whereas HLH occurred later (mean latency 35 days), often in the setting of established DRESS syndrome. Consistent with prior studies, antibiotic-associated cases demonstrated shorter latency to both DRESS and HLH onset [54,55]. Sequential presentation was more common than concurrent, and in more than half of the cases, HLH occurred after DRESS syndrome with or without initial improvement, which may be associated with a trigger-driven process and could represent a continuum of immune dysregulation, though causality cannot be established from this observational data. Interestingly, in 3 cases, HLH preceded DRESS syndrome, and the typical manifestations, such as eosinophilia and skin rash, appeared only after HLH was diagnosed and treated with IVIG (2/3 cases) [29].

### 4.3. Clinical Manifestations of DRESS/HLH Overlap Syndrome

DRESS syndrome typically presents with fever, a widespread cutaneous eruption—most commonly a morbilliform or maculopapular rash—lymphadenopathy, and multiorgan involvement, with cutaneous findings present in 88.9% of patients in our cohort. The liver is most frequently affected, followed by the kidneys and lungs, with less common involvement of the heart, central nervous system, and gastrointestinal tract [56,57,58]. Laboratory abnormalities include eosinophilia, atypical lymphocytosis, and leukocytosis or leukopenia. Demonstration of viral reactivation—particularly HHV-6, EBV, CMV, and HHV-7—is variable [59]. In contrast, HLH is a hyperinflammatory syndrome characterized by fever, hepatosplenomegaly, pancytopenia (including non-hemolytic anemia), and progressive multiorgan dysfunction, with laboratory findings including hyperferritinemia, hypertriglyceridemia, hypofibrinogenemia, elevated soluble IL-2 receptor levels, decreased or absent natural killer cell activity, and frequent hemophagocytosis on bone marrow examination, although its absence does not exclude the diagnosis [44]. Importantly, HLH in the absence of DRESS/HLH overlap syndrome does not usually present with eosinophilia, rash, lymphadenopathy, or facial edema.

In our cohort, hepatic involvement was observed in over 90% of patients, consistent with prior studies [60], while gastrointestinal involvement was rare but associated with severe outcomes, including one fatal case of enterocolitis [37]. Renal involvement occurred in approximately one-third of patients and was associated with a more severe clinical course, suggesting that kidney injury reflects systemic disease severity rather than isolated organ involvement. Mortality in this subgroup reached 38.5%, markedly higher than that reported in DRESS syndrome with kidney involvement alone [61]. In one patient, hematemesis and epistaxis were presenting symptoms, likely secondary to severe thrombocytopenia and coagulopathy, necessitating cryoprecipitate administration [23].

Clinicians should maintain a high index of suspicion for DRESS/HLH overlap syndrome in patients with suspected or confirmed DRESS syndrome who exhibit clinical deterioration, persistent or recurrent fever, or progressive organ dysfunction despite appropriate initial therapy, particularly corticosteroids. While DRESS syndrome typically follows a subacute course with improvement after withdrawal of the offending agent and initiation of immunosuppression, the recurrence of fever, development of worsening cytopenias, marked hyperferritinemia, coagulopathy, or rapidly evolving MOF should prompt evaluation for superimposed HLH. Conversely, in patients initially diagnosed with HLH, the persistence of fever, development of eosinophilia, lymphadenopathy, facial swelling, and a prominent cutaneous eruption should raise suspicion for evolving DRESS syndrome. Misdiagnosis is not uncommon and may delay appropriate treatment, resulting in increased mortality. Lack of expected clinical improvement or ongoing deterioration should therefore prompt early reassessment and consideration of an alternative or overlapping diagnosis.

The breadth of the differential diagnosis contributes directly to the 30.6% rate of initial misdiagnosis observed here (Table 3). Sepsis and severe bacterial or viral infection dominate, accounting for 81.8% of incorrect initial diagnoses, and are the most consequential mimics, because escalating antimicrobial therapy while the culprit drug is continued allows the syndrome to progress unchecked. Other entities that warrant consideration include primary Epstein–Barr virus or cytomegalovirus infection, adult-onset Still disease and systemic juvenile idiopathic arthritis, hemophagocytosis secondary to lymphoma or leukemia—one patient in our cohort was initially treated with CHOP chemotherapy for presumed lymphoma [10], and another was recognized only at autopsy in conjunction with Erdheim–Chester disease [26]—systemic lupus erythematosus, vasculitis, Stevens–Johnson syndrome/toxic epidermal necrolysis, acute generalized exanthematous pustulosis, isolated drug-induced liver injury, thrombotic microangiopathy, and, in endemic or immunosuppressed settings, disseminated tuberculosis, histoplasmosis, leishmaniasis, and cryptococcal disease. The practical discriminators in our data were eosinophilia (77.8%), a morbilliform eruption with facial edema, and lymphadenopathy (72.2%), which are characteristic of DRESS syndrome and uncommon in HLH of other causes, combined with marked hyperferritinemia (91.7%), progressive cytopenias, hypertriglyceridemia, and hypofibrinogenemia, which are typical of HLH and not expected in uncomplicated DRESS syndrome. Where both clusters are present simultaneously, the overlap syndrome should be assumed until disproved.

### 4.4. Treatment and Mortality in DRESS/HLH Overlap Syndrome

The cornerstone of DRESS syndrome management is immediate withdrawal of the culprit medication and supportive care. Systemic corticosteroids are commonly used, typically prednisolone 40–60 mg/day followed by a slow taper over at least 6–8 weeks to reduce the risk of relapse, although optimal dosing and duration remain unstandardized [3]. In refractory or steroid-resistant cases, adjunctive therapies such as cyclosporine, IVIG, or plasmapheresis may be considered [62,63,64,65]. In contrast, treatment of HLH is directed at rapidly controlling the underlying hyperinflammatory state while addressing the precipitating trigger. Standard approaches are based on HLH-94/HLH-2004 protocols and include high-dose dexamethasone (e.g., 10 mg/m^2^/day) with a structured taper over approximately 8 weeks, often in combination with etoposide [5,66]. Additional therapies, such as cyclosporine, may be used in selected cases, and hematopoietic stem cell transplantation is reserved for familial, persistent, or relapsing disease. The mean duration of the corticosteroid taper in our study was approximately 11 weeks, likely due to prolonged and more severe illness in patients with the overlap syndrome. Janus kinase (JAK) inhibitors, mepolizumab anti IL-5) have also been successfully used in DRESS syndrome cases, while Anakinra (anti IL-1) and emapalumab (anti IFN-γ) might be considered for refractory HLH cases. 

In our cohort, all patients received corticosteroids with the exception of one pediatric patient who improved following discontinuation of a suspected antibiotic trigger [41]. He was concurrently diagnosed with X-linked lymphoproliferative syndrome type 1, a primary immunodeficiency associated with significant immune dysregulation and increased risk of HLH. This favorable outcome suggests that early removal of the inciting agent may be sufficient to halt disease progression even in predisposed individuals. In contrast, a middle-aged woman with DRESS syndrome secondary to tocilizumab did not receive corticosteroids until progression to HLH was noted, at which point she developed steroid-refractory disease and the outcome was ultimately fatal [27]. Supportive care is critical, and several patients required prolonged hospitalization; notably, one patient remained hospitalized for over 150 days before ultimately succumbing to the illness [11].

The overall mortality rate in our cohort was 27.8% (Figure 3). Compared with DRESS syndrome alone, which carries a mortality of approximately 10% [43], HLH is associated with a substantially higher mortality, up to 60% [67]. Mortality in our study was higher among patients with steroid-refractory disease and those with MOF, in whom it approached 50%. These findings are both clinically and biologically plausible, as they likely reflect a higher degree of systemic immune dysregulation and cytokine-driven inflammation, possibly necessitating escalation of immunosuppressive therapy beyond corticosteroids. Delayed or missed diagnosis also appeared to contribute to worse outcomes. In one case, HLH was diagnosed only postmortem and identified on autopsy in conjunction with Erdheim–Chester disease [26]. In other cases, initial misdiagnoses—for example, lymphoma treated with CHOP chemotherapy [10]—or delayed initiation of corticosteroids for DRESS may have contributed to disease progression and adverse outcomes. We observed higher mortality rates among patients with sequential onset of DRESS and HLH, those exposed to non-antibiotic and non-antiepileptic medications, and those without recurrence during steroid taper. The latter may reflect a subgroup with more severe disease who did not achieve sufficient clinical improvement to undergo steroid tapering. The higher mortality observed among patients whose suspected trigger was neither an antibiotic nor an antiepileptic may reflect the relative under recognition of less common medication triggers. A lower clinical suspicion for DRESS associated with other drug classes could potentially contribute to delays in diagnosis and treatment, particularly in patients who develop DRESS/HLH overlap syndrome.

### 4.5. Practical Points for Clinical Practice

Several practical points follow directly from these results. First, suspect superimposed HLH whenever a patient with DRESS syndrome fails to improve: a sequential presentation accounted for 61.1% of cases, and clinical or laboratory worsening during corticosteroid therapy carried a mortality of 47.4% compared with 5.9% in corticosteroid-responsive patients. Second, reconsider the diagnosis rather than simply intensifying antimicrobial therapy; an initially incorrect diagnosis, most often presumed bacterial or viral infection, occurred in 30.6% of patients and was associated with more than double the mortality (45.5% vs. 20.0%). Third, measure ferritin, triglycerides, fibrinogen, soluble interleukin-2 receptor, and a full blood count in every patient with DRESS syndrome and internal organ involvement, since hyperferritinemia was present in 91.7% of this cohort and was the most consistent laboratory signal of overlap. Fourth, do not require bone marrow hemophagocytosis to make the diagnosis, as it was documented in only 63.9% of cases and its absence does not exclude HLH. Fifth, test for viral reactivation, documented in 38.9% of patients (Epstein–Barr virus, human herpesvirus 6, cytomegalovirus, and human herpesvirus 7), because reactivation identified patients who did poorly on corticosteroid monotherapy both in our cohort and in the earlier review. Sixth, escalate therapy early in patients with multiorgan failure, in whom mortality reached 53.8%: intravenous immunoglobulin (33.3%), etoposide (13.9%), and cyclosporine (5.6%) were reserved for the most severe cases, and the mean corticosteroid taper in this cohort was approximately 11 weeks, appreciably longer than that usually recommended for DRESS syndrome alone. Emerging targeted therapies, including JAK inhibitors, IL-1 inhibitors, IL-5/IL-5 receptor–targeted therapies, and IFN-γ blockade, may represent potential treatment options for severe-refractory DRESS/HLH overlap syndrome; however, their use remains supported primarily by limited case reports and small case series. Seventh, review the medication list with particular attention to antibiotics and antiepileptics, and withdraw the suspected agent promptly; withdrawal alone was sufficient in one pediatric patient, whereas delayed withdrawal or delayed initiation of corticosteroids preceded several deaths. Finally, in children, who comprised 27.8% of the cohort, pursue genetic evaluation for primary HLH even when a drug trigger is evident, since familial disease was rarely excluded in the source reports.

### 4.6. Limitations

This study has several limitations. First, the sample size was small, reflecting the rarity of DRESS–HLH overlap syndrome, which limited the ability to perform robust statistical analyses and draw definitive conclusions. Given the small sample size, formal statistical comparisons between subgroups were not performed. As a result, the findings are primarily descriptive and should be interpreted with caution. The retrospective nature of data collection further limits causal inference. Second, the data were derived largely from case reports and case series, introducing potential publication bias and heterogeneity in clinical presentation, diagnostic evaluation, and management. Publication bias likely inflates the observed mortality rate and may skew the apparent predominance of certain triggers, since fatal or clearly attributed cases are more likely to be published. Important variables were inconsistently reported across studies, and missing data may have led to underestimation of certain clinical features. Additionally, this review was not registered in PROSPERO or another registry, and our search was restricted to English-language publications, as our team was unable to verify non-English reports without translation. Despite these limitations, this study provides a comprehensive descriptive synthesis of a rare and clinically important DRESS/HLH overlap syndrome.

### 4.7. The Need for Multicenter Collaboration

DRESS/HLH overlap syndrome is genuinely rare. An exhaustive search of three databases from inception through March 2026 yielded only 36 patients reported worldwide, distributed across 28 articles and 5 conference abstracts, a median of one patient per publication. No prospective cohort, randomized trial, or dedicated registry exists, the true incidence is unknown, and no single center is likely to accumulate enough cases to generate one. This scarcity is the principal barrier to progress: it precludes formal statistical comparison between subgroups, prevents adjustment for confounding, and makes it impossible to determine whether intravenous immunoglobulin, etoposide, or cyclosporine confers benefit over corticosteroids alone. Answering these questions will require multicenter and international collaboration, ideally through a shared prospective registry with a standardized data dictionary capturing the individual components of the RegiSCAR score and HScore, systematic viral reactivation testing, HLA and cytotoxicity genotyping, the sequence and dosing of immunosuppressive therapy, and long-term outcomes. Established severe cutaneous adverse reaction networks, together with existing HLH consortia, provide a natural framework for such an effort. Until such a registry exists, we encourage authors of future case reports to document the full set of variables outlined here, so that individual-patient-data synthesis remains feasible.

## 5. Conclusions

DRESS/HLH overlap syndrome is characterized by profound immune dysregulation and systemic hyperinflammation, most commonly occurring in a sequential pattern in which DRESS syndrome precedes secondary HLH. The two may represent points along a shared pathophysiologic continuum, with HLH reflecting a more severe end of immune activation. This overlap syndrome carries a mortality rate higher than DRESS syndrome alone and approaching that reported for HLH. Antibiotics were the most commonly implicated medication class, although the causative agent was not identified in a subset of cases, highlighting the need for more reliable diagnostic strategies to determine the offending drug. Mortality was associated with diagnostic delay: in approximately 30% of cases, the syndrome was initially mistaken for bacterial or viral infection. In our cohort, steroid-refractory disease and multiorgan failure were also associated with worse clinical outcomes and increased mortality. These findings underscore the importance of early recognition and timely therapeutic escalation in high-risk patients. Further research is needed to identify specific biomarkers across this disease spectrum and to better define factors that influence clinical outcomes. Given the rarity of this syndrome, such research will only be feasible through multicenter and international collaboration built around a shared prospective registry.

## Figures and Tables

**Figure 1:**
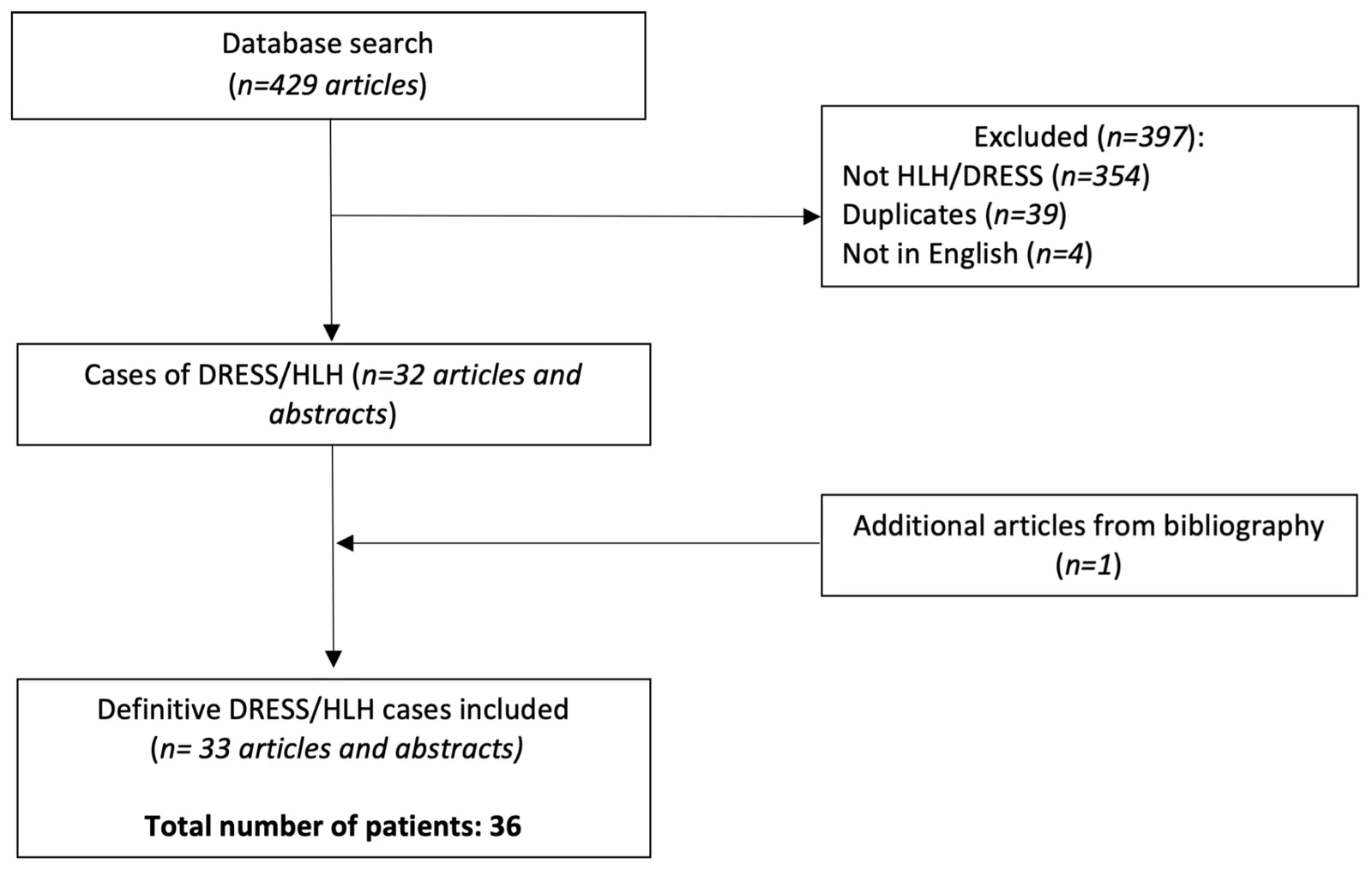
PRISMA flow diagram of study selection.

**Figure 2:**
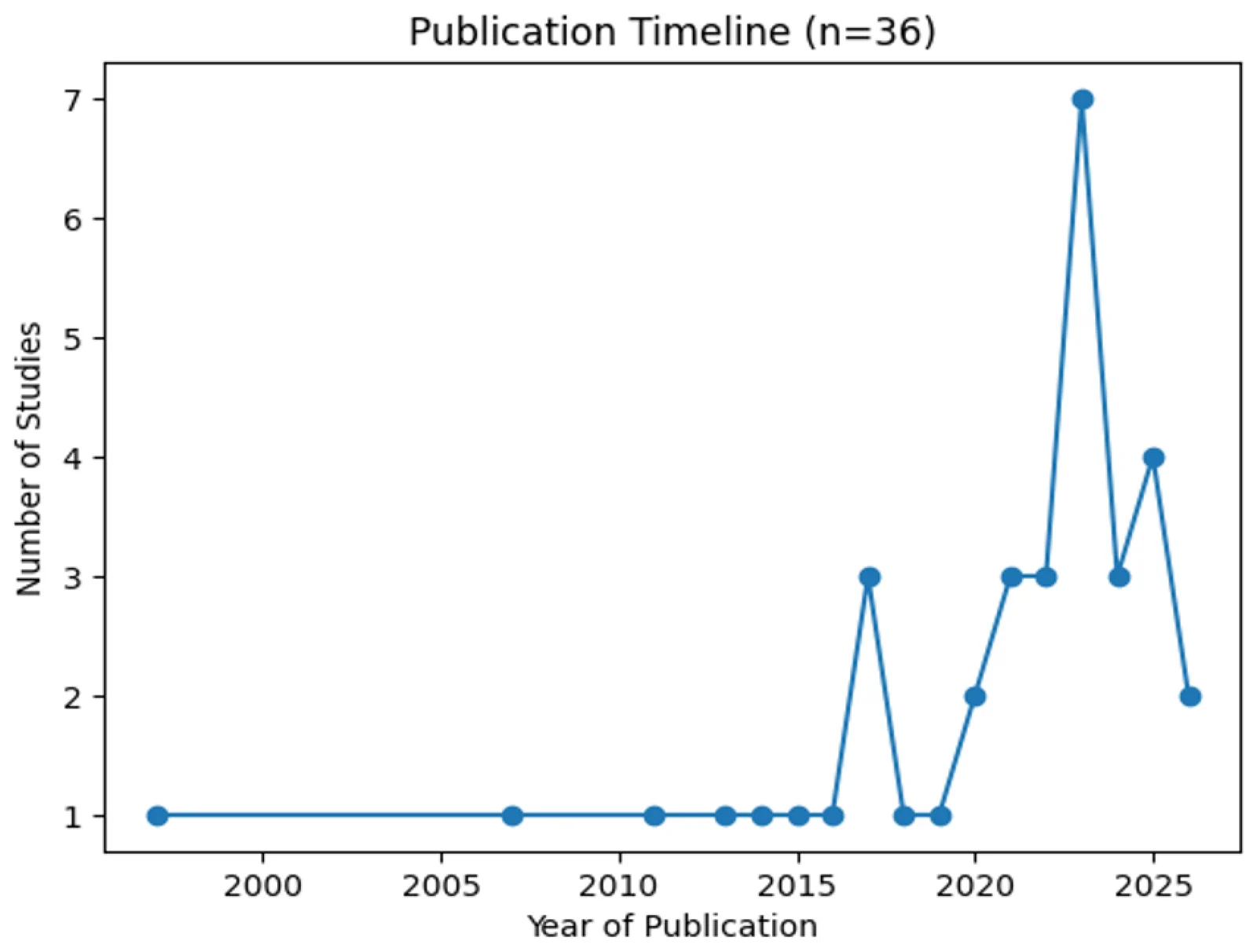
Year of publication of included studies.

**Figure 3:**
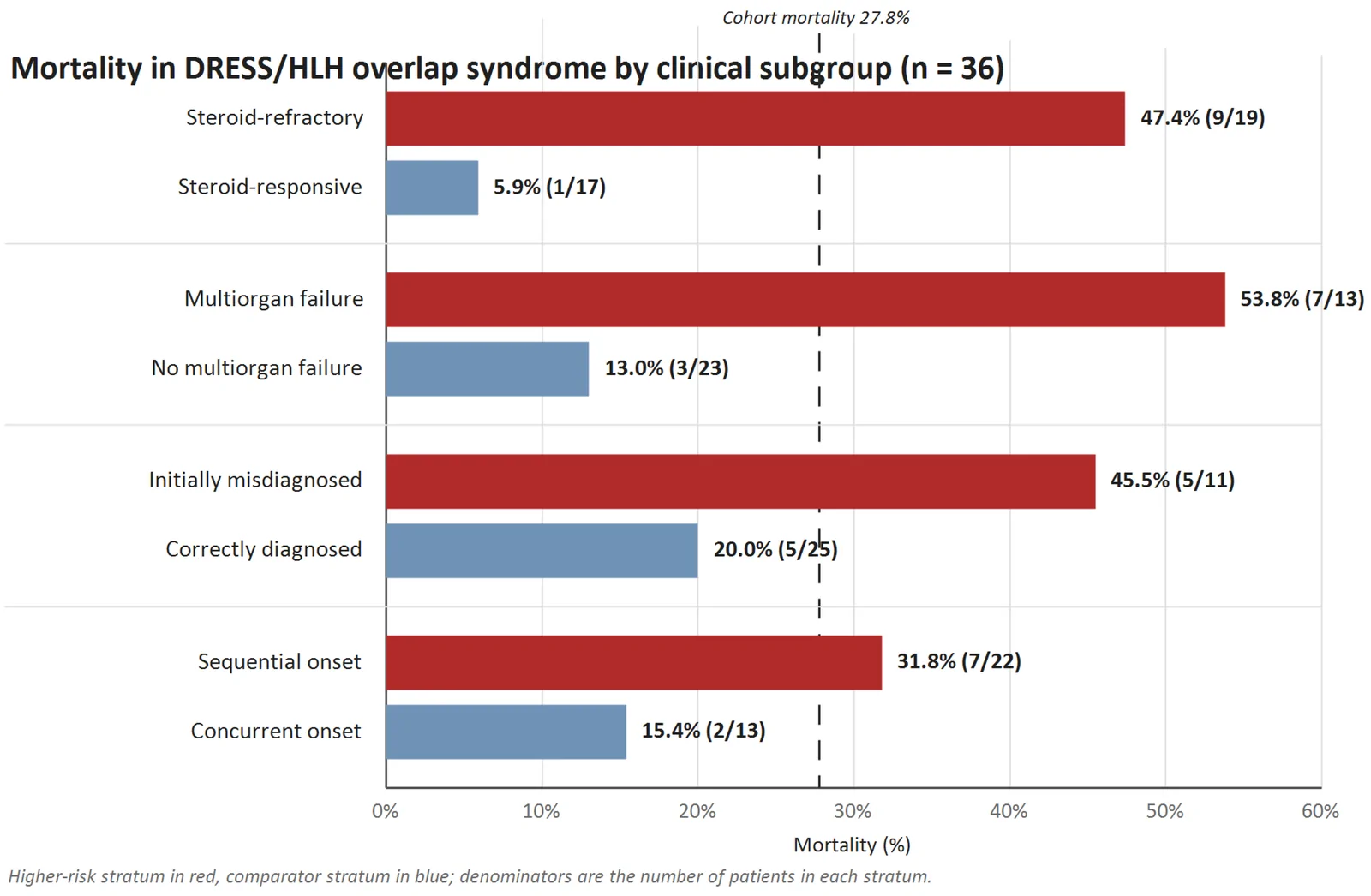
Mortality in DRESS/HLH overlap syndrome stratified by clinical subgroup. Bars show the proportion of patients who died within each stratum, with the number of deaths and the number of patients in that stratum given alongside. The dashed line marks the overall cohort mortality of 27.8% (n = 10/36). Steroid-refractory disease was defined as clinical or laboratory worsening during corticosteroid therapy. Because of the small subgroup sizes, these comparisons are descriptive and were not tested statistically.

**Table 1:** Cohort characteristics of patients with DRESS/HLH overlap syndrome (n = 36).

Variable	Mean (SD or Range)	N (%)
Age, years	37.3 (21.8)	
Gender—male		9 (25.0)
Gender—female		27 (75.0)
Pediatric (<18 years of age)		10 (27.8)
Medication group		
Antibiotics		13 (36.1)
Antiepileptics		9 (25.0)
Other *		14 (38.9)
Latency, DRESS (days)	27.9 (3–179)	
Latency, HLH (days)	35.0 (3–160)	
Viral reactivation **		14 (38.9)
EBV		6 (16.7)
HHV-6		6 (16.7)
CMV		2 (5.6)
HHV-7		1 (2.8)
Visceral organ involvement		
Liver		34 (94.4)
Kidney		13 (36.1)
Esophagus, pancreas, colon, CNS, heart, lungs		1 each (2.8)
Laboratory characteristics		
Hyperferritinemia		33 (91.7)
Eosinophilia		28 (77.8)
Anemia		27 (75.0)
Thrombocytopenia		27 (75.0)
Lymphadenopathy		26 (72.2)
Hemophagocytosis on bone marrow		23 (63.9)
Hypertriglyceridemia		14 (38.9)
Hypofibrinogenemia		14 (38.9)
Leukopenia		13 (36.1)
Splenomegaly		13 (36.1)
High soluble IL2-R		9 (25.0)
Low/absent NK cell activity		6 (16.7)
Treatment		
Steroids		35 (97.2)
IVIG		12 (33.3)
Etoposide		5 (13.9)
Cyclosporine		2 (5.6)
Multiorgan failure		13 (36.1)
ICU admission		7 (19.4)
Secondary infection		4 (11.1)
Mortality ***		10 (27.8)
Mortality, correct initial diagnosis		5/25 (20.0)
Mortality, initially incorrect diagnosis		5/11 (45.5)

* Other implicated medications included leflunomide, sulfasalazine, allopurinol, hydroxychloroquine, tocilizumab, and Boswellia serrata (nutritional supplement); in 4 cases (11.1%) an antibiotic was suspected but not specified, and in 4 cases (11.1%) the culprit medication was unknown. ** Viral reactivation percentages reflect individual viruses among the 14 patients (38.9%) with documented reactivation. *** Mortality stratified by temporal pattern (concurrent vs. sequential) is presented in Table 2. DRESS—Drug Reaction with Eosinophilia and Systemic Symptoms; HLH—Hemophagocytic Lymphohistiocytosis; EBV—Epstein–Barr virus; HHV—human herpesvirus; CMV—cytomegalovirus; CNS—central nervous system; NK—natural killer; IL2-R—interleukin-2 receptor; IVIG—intravenous immunoglobulin; ICU—intensive care unit.

**Table 2:** Temporal relationship between DRESS syndrome and HLH and associated mortality.

Temporal Pattern	N (%)	Mortality, n/N (%)
Concurrent	13 (36.1)	2/13 (15.4)
Sequential	22 (61.1)	7/22 (31.8)
DRESS → HLH	10 (27.8)	3/10 (30.0)
DRESS → initial improvement → HLH	9 (25.0)	3/9 (33.3)
HLH → initial improvement → DRESS	2 (5.6)	1/2 (50.0)
HLH → DRESS	1 (2.8)	0
Unknown	1 (2.8)	1/1 (100)

DRESS—Drug Reaction with Eosinophilia and Systemic Symptoms; HLH—Hemophagocytic Lymphohistiocytosis.

**Table 3:** Differential diagnosis of DRESS/HLH overlap syndrome.

Alternative Diagnosis	Features Favoring the Alternative Diagnosis	Features Favoring DRESS/HLH Overlap	Most Useful Discriminating Tests
Sepsis or severe bacterial infection	Hypotension, neutrophilia with left shift, elevated procalcitonin, positive cultures, no eosinophilia	Eosinophilia, morbilliform eruption with facial edema, new drug started 2–6 weeks earlier, ferritin often >10,000 ng/mL	Blood cultures, procalcitonin, absolute eosinophil count, careful drug timeline
Primary EBV or CMV infection	Younger patient, pharyngitis, atypical lymphocytosis, self-limited course	Drug exposure precedes onset; extensive eruption; marked hyperferritinemia; progressive organ failure	EBV and CMV serology with quantitative PCR (reactivation is itself common in DRESS syndrome)
Adult-onset Still disease or systemic JIA	Evanescent salmon-colored rash, arthritis, sore throat, no drug trigger	Identifiable culprit drug, eosinophilia, facial edema, lymphadenopathy	Yamaguchi criteria, rash morphology, drug history
Lymphoma- or leukemia-associated HLH	Bulky or persistent lymphadenopathy, B symptoms, cytopenias without eosinophilia	Eosinophilia, cutaneous eruption, clear temporal link to a drug	Lymph node and bone marrow biopsy, PET-CT
Stevens–Johnson syndrome/toxic epidermal necrolysis	Painful skin, mucosal erosions, epidermal detachment, positive Nikolsky sign	Facial edema, eosinophilia, visceral involvement without epidermal detachment	Skin biopsy, body-surface-area detachment, mucosal examination
Acute generalized exanthematous pustulosis	Onset within 4 days of exposure, sterile pustules on erythema, neutrophilia	Latency of 2–6 weeks, eosinophilia, hepatitis, hyperferritinemia	Skin biopsy, latency from drug exposure
Isolated drug-induced liver injury	Transaminitis without cytopenias, normal ferritin and fibrinogen, usually afebrile	Cytopenias, hyperferritinemia, hypofibrinogenemia, splenomegaly	Complete blood count, ferritin, triglycerides, fibrinogen
Systemic lupus erythematosus or vasculitis	Chronic course, nephritis, serositis, characteristic autoantibodies	Acute drug-timed onset, eosinophilia, marked hyperferritinemia	ANA, ANCA, complement levels, urinalysis
Thrombotic microangiopathy	Schistocytes, very high LDH and D-dimer renal failure, no rash	Morbilliform eruption, eosinophilia, hemophagocytosis on marrow	Blood film, ADAMTS13 activity, haptoglobin
Disseminated opportunistic infection (tuberculosis, histoplasmosis, leishmaniasis, cryptococcosis)	Endemic exposure or immunosuppression; may itself precipitate secondary HLH	Identifiable culprit drug, eosinophilia, cutaneous eruption preceding infection	Cultures, antigen testing, bone marrow examination, cross-sectional imaging

DRESS—Drug Reaction with Eosinophilia and Systemic Symptoms; HLH—Hemophagocytic Lymphohistiocytosis; EBV—Epstein–Barr virus; CMV—cytomegalovirus; JIA—juvenile idiopathic arthritis; LDH—lactate dehydrogenase; ANA—antinuclear antibody; ANCA—antineutrophil cytoplasmic antibody; PET-CT—positron emission tomography–computed tomography; ADAMTS13—a disintegrin and metalloproteinase with a thrombospondin type 1 motif, member 13.

## Data Availability

The data supporting the findings of this study were extracted from the previously published articles and conference abstracts cited in the References Section. No new datasets were generated for this study.

## Supplementary Materials

The following supporting information can be downloaded at https://www.mdpi.com/article/10.3390/jcm15197383/s1, PRISMA 2020 Checklist.

## Abbreviations

The following abbreviations are used in this manuscript:

DRESS	Drug Reaction with Eosinophilia and Systemic Symptoms
HLH	Hemophagocytic Lymphohistiocytosis
PRISMA	Preferred Reporting Items for Systematic Reviews and Meta-Analyses
RegiSCAR	Registry of Severe Cutaneous Adverse Reactions
HScore	Hemophagocytic Syndrome Diagnostic Score
EBV	Epstein–Barr Virus
CMV	Cytomegalovirus
HHV-6	Human Herpesvirus 6
HHV-7	Human Herpesvirus 7
CNS	Central Nervous System
NK	Natural Killer (cells)
IL-2	Interleukin-2
IL2-R	Interleukin-2 Receptor
IVIG	Intravenous Immunoglobulin
ICU	Intensive Care Unit
MOF	Multiorgan Failure
